# Two Rounds of Whole Genome Duplication in the Ancestral Vertebrate

**DOI:** 10.1371/journal.pbio.0030314

**Published:** 2005-09-06

**Authors:** Paramvir Dehal, Jeffrey L Boore

**Affiliations:** **1**Evolutionary Genomics Department, Department of Energy Joint Genome Institute and Lawrence Berkeley National Laboratory, Walnut Creek, California, United States of America,; **2**Department of Integrative Biology, University of California, Berkeley, California, United States of America; University of OxfordUnited Kingdom

## Abstract

The hypothesis that the relatively large and complex vertebrate genome was created by two ancient, whole genome duplications has been hotly debated, but remains unresolved. We reconstructed the evolutionary relationships of all gene families from the complete gene sets of a tunicate, fish, mouse, and human, and then determined when each gene duplicated relative to the evolutionary tree of the organisms. We confirmed the results of earlier studies that there remains little signal of these events in numbers of duplicated genes, gene tree topology, or the number of genes per multigene family. However, when we plotted the genomic map positions of only the subset of paralogous genes that were duplicated prior to the fish–tetrapod split, their global physical organization provides unmistakable evidence of two distinct genome duplication events early in vertebrate evolution indicated by clear patterns of four-way paralogous regions covering a large part of the human genome. Our results highlight the potential for these large-scale genomic events to have driven the evolutionary success of the vertebrate lineage.

## Abstract 2

The authors compared genomic map positions of paralogous genes from four vertebrate genomes and found evidence for two distinct genome duplication events in the vertebrate lineage.

## Introduction

It has long been hypothesized that the increased complexity and genome size of vertebrates has resulted from two rounds (2R) of whole genome duplication (WGD) occurring in early vertebrate evolution, thus providing the requisite raw materials [1]. This seemed to be supported by the long-standing speculation that humans have about 100,000 genes, roughly four times the number expected for invertebrates' genomes, but this is now known to be incorrect, with the actual human gene count being closer to 30,000 [2,3]. Conflicting analyses have now made this very controversial, with some studies supporting 2R (e.g., [4–8]), others seeing only a single round of WGD (e.g., [9–11]), and still others refuting WGD altogether by concluding that nothing greater than limited segmental duplications have occurred (e.g., [12,13]).

The 2R hypothesis had been bolstered by observations that a few gene families, e.g., Hox clusters [14], follow a “4:1 rule” in the numbers of vertebrate to invertebrate genes. However, comparison of the complete genome sequences of human [2,3] and *Drosophila* [15] revealed that less than 5% of homologous gene families follow the 4:1 rule [12]. Further, although two sequential duplications are expected to generate the evolutionary topology (AB)(CD) for the descendent genes, rather than (A)(BCD), in fact, the relationships of vertebrate multigene families do not generally show this pattern, as indicated by early studies using only a few genes [16] and confirmed as complete genome sequences became available [2,13]. (However, for a different view, see [17].) Several studies have incorporated data from sparse sampling of genes from taxa thought to have branched near to these purported duplications, including lamprey [18], hagfish, amphioxus [17,19–22], and *Ciona* [23]; although these results are useful for timing duplications, the conclusions could never be viewed as definitively resolving this issue because these products could have alternatively been generated by duplications of individual genes or short gene segments rather than by WGDs. Even duplicating all of the genes in a genome individually is quite different from a whole genome duplicating simultaneously.

There are several reasons why this has been a difficult issue to resolve. After duplication, only the minority of gene pairs will adopt a new function (“neofunctionalization”) or partition old functions (“subfunctionalization”) quickly enough to escape disabling mutations that would lead to their eradication [24]; therefore, rampant gene loss rapidly erases this signal of genome duplication. Further, four-member gene families, even those with the (AB)(CD) topology, can be generated by two rounds of duplications of individual genes or of segments much smaller than the entire genome, generating a condition that cannot be differentiated on this basis from 2R followed by many gene losses. This alternative scenario seems especially plausible because recent analyses have shown that gene duplications occur much more frequently than had been thought, with the typical rate being sufficient to duplicate an entire genome equivalent every 100 million years (MY) [25,26]. Until recently, no complete genome sequence has been available from an outgroup that is closely related to vertebrates, and all methods of phylogenetic reconstructions are less accurate with more distant relatives such as *Drosophila* and yeast [20]. Lastly, there has not been to date a method to accurately and comprehensively cluster genes into homologous families because methods that rely on sequence similarity alone are highly subject to artifactual association of slowly evolving paralogs and to erroneous exclusion of the more rapidly evolving genes.

**Figure 1:**
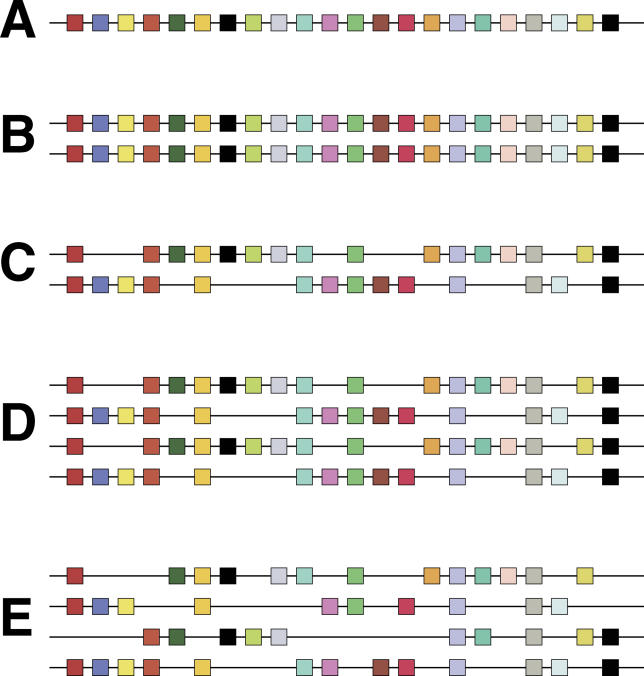
Pattern Predicted for the Relative Locations of Paralogous Genes from Two Genome Duplications (A) Representation of a hypothetical genome that has 22 genes shown as colored squares. (B) A genome duplication generates a complete set of paralogs in identical order. (C) Many paralogous genes suffer disabling mutations, become pseudogenes, and are then lost. One could imagine this condition being evidence of a single round of genome duplication followed by significant gene losses. (D) A second genome duplication recreates another set of paralogs in identical order, with multigene families that retained two copies now present in four, and those that had lost a member now present in two copies. (E) Again, many paralogous genes suffer disabling mutations, become pseudogenes, and are then lost. Of course, unrelated gene duplications and transpositions can occur. Even though this leaves only a few four-member gene families, the patterns of 2- and 3-fold gene families unite, in various combinations, all four genomic segments, revealing that the sequential duplications had been of very large regions, in this case all or nearly all of this hypothetical genome.

Fortunately, as has been shown convincingly for the yeast genome and for *Arabidopsis* [27–30], evidence of an ancient genome duplication can be seen in the large-scale pattern of the physical locations of homologous genes, even when the great majority of the duplicated genes have been lost. Studies have shown that the human genome also has multiple regions of colinear paralogous gene copies [4,21,22,31–37], but considered the arrangements of too small a number of genes and genomic regions to be comprehensive. This approach is now available for a large-scale evaluation of the vertebrate 2R hypothesis, because complete (i.e., at least draft quality) genome sequences are available for the tunicate *Ciona intestinalis* [38] (a basal chordate outgroup) and the vertebrates *Takifugu rubripes* [39] (a pufferfish “fugu”), *Mus musculus* [40] (mouse), and human [2,3]. Figure 1 illustrates how the signal of two rounds of genome duplication could be retained by the large-scale pattern in location of duplicated genes, in which many tracks of paralogous duplicates (which may not contain identical subsets of genes) each occur at exactly four positions in the genome, i.e., “tetra-paralogons.” No similar signal would be generated by repeated duplications of genes or even large gene segments; only WGDs would result in such global organization of paralogous genes.

## Results

### Gene Clustering and Duplication Timing

**Figure 2:**
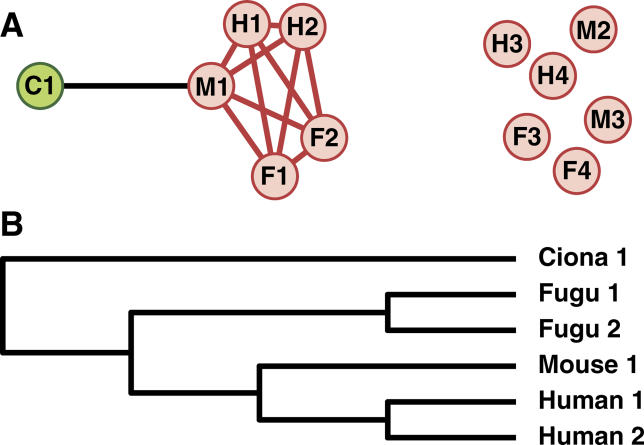
Overview of the Building of a Gene Cluster and Phylogenetic Tree Shown by a Hypothetical Example (A) Each circle represents a gene, labeled with the source genome according to the first letter of each taxon—C, M, H, and F for *Ciona,* mouse, human, and fugu, respectively—and further differentiated by numeral. BLASTP was first used to search all vertebrate genes for the one most similar to *Ciona'*s *C1* gene, in this case the mouse gene *M1.* Then other genes are recruited to the cluster if they have a higher similarity score to *M1* that that between *C1* and *M1,* indicated here by the red lines. The six genes shown on the right side of the diagram have some sequence similarity to those in the cluster, but less than that between *C1* and *M1,* so are not included. Because the vertebrates are more closely related to each other than any is to *Ciona,* each cluster will include those genes descended from a single gene in the common chordate ancestor, having arisen by either lineage splitting or gene duplication specific to one or more vertebrates. (See Materials and Methods for more details.) (B) Evolutionary tree of the genes in this cluster show separate duplications for fugu and for human. Because the maximum likelihood method does not rely solely on sequence similarity, there is no significance to the mouse gene being most similar to *C1.* The mouse genome simply contained the most slowly evolving vertebrate gene in this multigene family; this can be from any vertebrate taxon with approximately equal likelihood.

**Figure 3:**
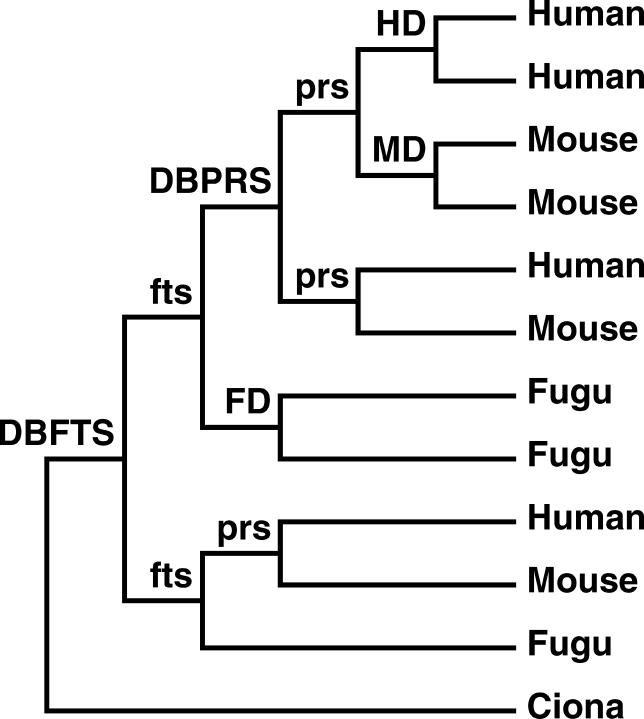
Hypothetical Phylogenetic Tree Showing All Possible Types of Gene Relationships and How They Are Most Parsimoniously Interpreted Interior nodes are designated in lower case for those that simply result from lineage splitting and in upper case for gene duplications within a lineage. Although not shown, nodes are still scored if there is gene loss. Phylogenetic trees for each gene family can be viewed at http://phigs.org/, also providing a valuable tool for improving the inference of gene function. DBFTS, duplication before fish–tetrapod split; DBPRS, duplication before primate–rodent split; FD, fugu duplication; fts, fish–tetrapod split; HD, human duplication; MD, mouse duplication; prs, primate–rodent split.

A graph-based method was used with the complete gene sets of the four chordates (98,517 total genes; see Table 1 for details of each step in the analysis) to generate clusters such that each contains all, and only, those genes that descended from a single gene in their common ancestor (Figure 2). A multiple sequence alignment and a maximum likelihood evolutionary tree were constructed for each cluster, then a Web browser interface was built so that each can be viewed individually. (For more details and updates that include more taxa, see the “PhIGs” [Phylogenetically Inferred Groups] Web site at http://phigs.org/.) We could then easily determine when each gene duplicated relative to lineage splitting by comparing these gene trees with the known evolutionary relationships of the animals. For example, a gene duplication that is specific to only one animal's lineage is seen as two genes from the same genome clustered together. A gene that duplicated once in the unique common ancestor of mouse and human would generate a tree that groups gene copy 1 of human and mouse and, separately, gene copy 2 of human and mouse. Put more generally, gene duplications that are shared by more that one species are seen as a replication of the phylogeny of the descendant organisms for each gene copy. Of course, gene losses and various combinations of these processes are seen as well. Figure 3 shows all possible gene topologies along with how each would be interpreted.

**Table 1:**
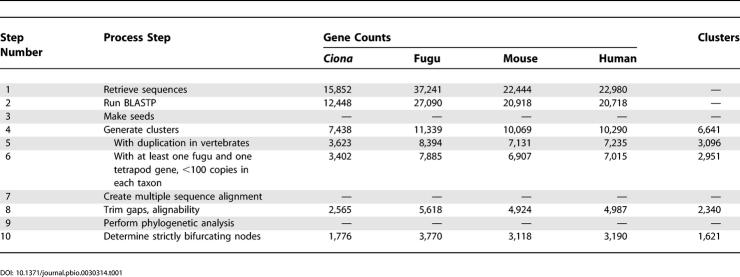
Overview of the Process for Analyzing the Complete Gene Sets with Number of Genes Included at Each Step

**Figure 4:**
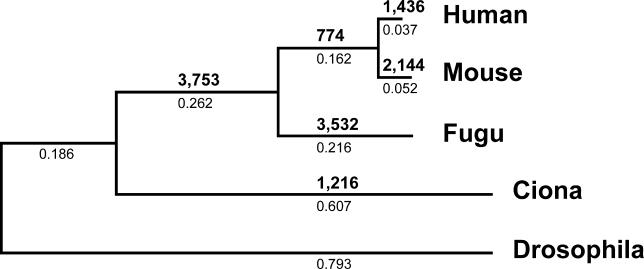
Phylogenetic Analysis of the Four Chordates with *Drosophila* as an Outgroup This phylogenetic tree is based on 766 concatenated single copy protein sequences totaling 313,797 amino acid positions with branches proportional to the amount of change. Numerals in bold above the branches indicate the number of gene duplications occurring in each lineage; numerals below indicate branch lengths.

This reveals that 46.6% of the ancestral chordate genes appear in duplicate in one or more of the vertebrate lineages, with 34.5% having at least one duplication before the divergence of fish from tetrapods and 23.5% having at least one duplication afterward. (Some of these are counted twice, having had duplications both before and after the fish–tetrapod split.) This means that there are 3,753 gene duplications placed at the base of Vertebrata, which is remarkable because the ancestral genome would be reasonably estimated to have had fewer than 20,000 genes, which is the case for the tunicate as well as other invertebrate outgroups. However, as can be seen in Figure 4, gene duplications are in large numbers on every branch of the tree, making it unclear whether this, in itself, indicates a significant acceleration in duplication rate. Additionally, of the gene clusters with duplications basal to the fish–tetrapod split, 20.6% have had one duplication event, 10.8% have had two, and 5.1% have had more than two, counter to the expectation from 2R, and casting further doubt on the significance of this for the 2R hypothesis.

### Gene Family Membership

An early observation in support of 2R was that several gene families have expanded from a single member in invertebrates to having four members for some vertebrates. Previous studies, confirmed in this analysis, have shown that this is not generally true for vertebrate multigene families [12]. As can be seen in Figure S1, there is no peak at four for gene family membership for any vertebrate. In fact, even gene duplications do not predominate; for each vertebrate species considered individually, one member per cluster is the largest category, accounting for 55%, 57%, and 59% of the fugu, mouse, and human genes, respectively, with 53.4% of the gene clusters having no duplication events whatsoever. Thus, there is no signal of 2R remaining in gene family membership, despite early anecdotal observations to the contrary.

### Determination of Concordantly Duplicated Regions

**Figure 5:**
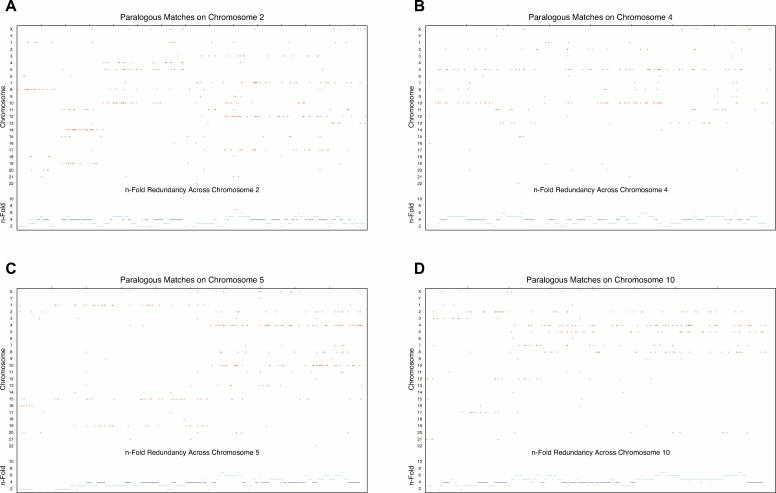
Plot of the Genomic Positions of Paralogous Pairs of Human Genes that Arose from Duplications Predating the Fish–Tetrapod Split The queries shown here use Chromosomes 2, 4, 5, and 10, as indicated for the four panels. (The complete set can be seen in Figure S2.) On the *x-*axis is each chromosome arranged from p to q telomeres. On the *y-*axis is each of the 22 human autosomes plus the X and Y chromosomes. For each query gene on the *x-*axis, a “hit” is scored if the subject chromosome contains a paralog generated by a gene duplication prior to the fish–tetrapod split. The lower portion of each panel plots the *n-*fold redundancy along the query chromosome as defined by pairs of paralogs detected in a sliding window analysis. See the Material and Methods section for details, but briefly, for every human query gene, a window was considered of 50 genes to the left and 50 genes to the right, with a “hit” obtained for the subject chromosome if it includes the early-duplicated paralogs of genes on each side of the query. Four-fold (i.e., including the query) matching, as expected by the 2R hypothesis, is highlighted in a darker shade of blue.

**Figure 6:**
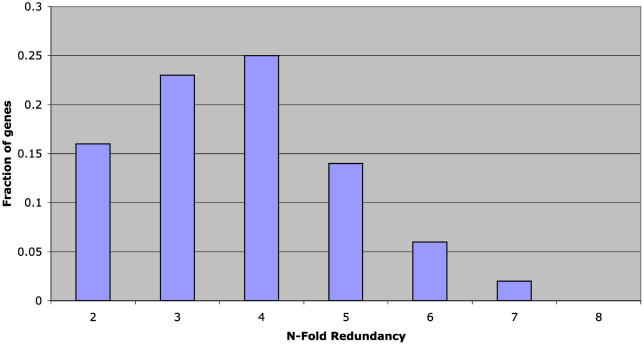
Histogram Showing the Lower Bound Estimate of *N-*fold Redundancy Using the Analysis Reported in Figure 5 This histogram is generated by counting the depth of paralogon redundancy across all human chromosomes as shown in the lower part of Figure S2 (and subsampled for Figure 5). The peak at 4-fold coverage is consistent with the 2R hypothesis, and constitutes a lower bound estimate, because the sliding window examines only a small span of flanking genes and would be highly subject to effects of local gene rearrangements.

To test the extent to which the 3,753 early duplication events that are timed to the base of Vertebrata were generated as part of larger scale, multigene duplications, we examined the relative positions of these resulting paralogs in the human genome (which is currently the best assembled and annotated vertebrate genome). These results are shown in Figure 5 (and more comprehensively in Figure S2) in which the linear array of genes for each chromosome is used to query for paralogs generated by any duplication event prior to the fish–tetrapod split. It is apparent from these figures that there is a large-scale pattern of genome segments that are concordant in having similar arrangements of paralogous genes. We quantified this by identifying all cases in which two or more different early-duplicating genes are within a 100-gene window, then, for each, querying all other places in the genome, using a sliding window to count the number of cases in which their respective paralogs are within both 50 genes upstream as well as 50 genes downstream from that point. There is a distinct pattern of having multiple chromosomes matching with long linear stretches of paralogous genes. This indicates that these duplications occurred in very large segments, consistent with the hypothesis of WGD(s). Having matches to three other chromosomal segments is the dominant category, as can be seen by the darker coloring in Figures 5 and S2 and in the histogram of Figure 6. These patterns, with each genomic region corresponding in gene arrangement to sets of paralogs in three other genomic segments, are strong support for the 2R hypothesis.

Although the 4-fold (i.e., including the query segment) category is the most prevalent, it accounts for only 25% of the genome. Nonetheless, it is striking that this remains the largest category despite approximately 450 MY of evolution. This constitutes a strong signal of 2R, and could not reasonably have been generated by a series of smaller duplication events. For the latter to have generated this pattern, multiple duplications of the same region (or its resulting duplicates) would have to have occurred three times, and have done so for many regions throughout the genome. We would expect, rather, that independent, random duplications would follow a Poisson distribution; this contrasting situation is seen when the same analysis is done with all human gene paralogs generated by duplication after the split of fish and tetrapods (not shown). Even if we were to consider the alternative of a single WGD followed by subsequent independent duplications of large segments, it would be difficult to explain why these would have been predominantly 2-fold for previously duplicated regions. The most parsimonious explanation for the observed pattern can only be 2R.

### Tetra-Paralogon Detection

To further establish 2R, we evaluated these sets of paralogs for whether this 4-fold matching indicates that they fall into tetra-paralogons, as illustrated in Figure 1. We formalized this by first identifying paralogons (paralogous genomic segments) containing the same set of at least two duplicated gene pairs, while allowing for a maximum of 100 unduplicated genes in between (similar to the approach in [10]). (The allowance of 100 genes is arbitrary, but the results are not critically dependent on this number, which is only used to find the blocks of paralogous genes.) We infer that duplicated genes in paralogons are likely to have arisen from a single duplication involving all contained, duplicated genes, and that the unique, intervening genes have resulted from differential gene deletions and subsequent genome rearrangements.

**Figure 7:**
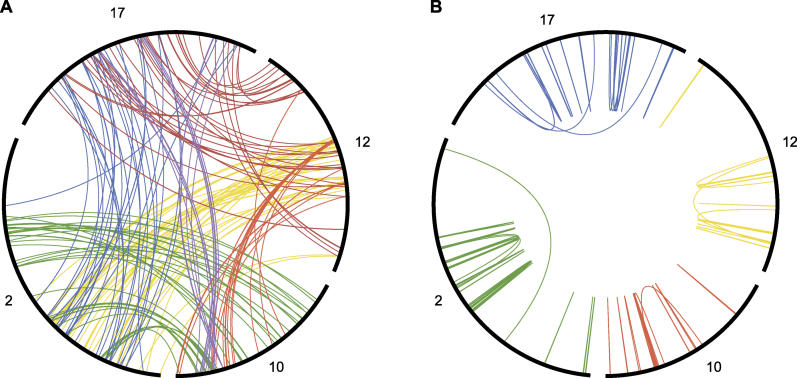
An Arbitrarily Selected Subset of the Human Genome Showing the Physical Relationships Among Paralogous Genes (A) This is an example of the tetra-paralogous relationships of a subset of human genes that are all inferred, by gene trees, to have duplicated prior to the split of fish from tetrapods, but after the split of *Ciona* from vertebrates. These genes are on four chromosomes with their identities indicated outside of the circle. The complete set of tetra-paralogons can be viewed in Figure S3. (B) In contrast, paralogous human genes generated by duplications after the split of fish and tetrapods, as shown for this sample of the same four human chromosomes, do not form such tetra-paralogons. Their pattern appears to result from smaller-scale tandem duplications of individual genes or segments, followed by slow rearrangements. In addition to these apparently functional gene pairs shown in the figure for this portion of the human genome, we have identified eight pseudogene pairs that occur on different chromosomes; it is not clear whether these pseudogenes are the result of random retrotransposition (or other rearrangement mechanisms) rather than gene conversion events between older duplicates, which would make it appear as though these had duplicated later than they actually did, as has been observed in yeast [29].

We identified 2,953 paralogous human gene pairs that are inferred to have resulted from 1,912 genes that duplicated prior to the divergence of the fish and tetrapod lineages (with some gene losses also). Of these paralogous genes, 32.4% are still in 386 detectable paralogons comprising 772 individual genomic segments, containing from two to 42 gene pairs (Table S1). Of these 772 genomic segments, 454 comprise tetra-paralogons (Figures 7A and S3, Table 2) as shown hypothetically in Figure 1, in which overlapping sets of paralogs fall into 4-fold groups. (Unfortunately, it was not possible for us to evaluate the hypothesis of an additional genome duplication unique to ray-finned fish [41,42] because of the generally poor contiguity of the fugu draft assembly.)

**Table 2:**
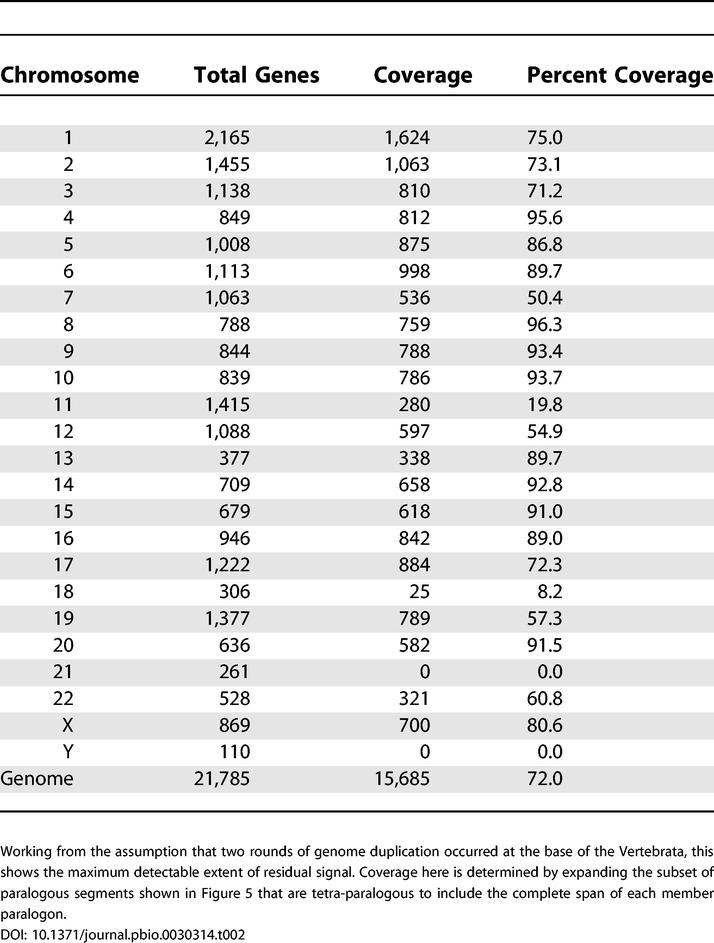
Distribution of the Human Genome's Tetra-Paralogons by Chromosome under the Most Permissive Model for Signal Detection

In contrast, when looking at the gene pairs that arose from a duplication event after the divergence of the fish and mammal lineages (see Figure 4), we find only 11% are detected in paralogons in the human genome, indicating that these duplications have less commonly included large segments of the genome (Figure 7B). This is especially interesting in that their relative recency would make it more likely that any large duplications would remain detectable, reinforcing the contrast with the large-scale structure of those earlier duplications. By looking specifically for tandemly duplicated genes by defining them as paralogs on the same chromosome that are separated by fewer than 10 intervening genes, we can recognize that 50% of these human gene pairs arose from tandem duplication, compared with 6% for the human gene pairs that arose before the divergence of the fish and tetrapod lineages.

## Discussion

No detectible signal of WGD exists in the analysis of gene family membership. There is no peak at four genes per family for any of the vertebrates (Figure S1) as might result from 2R. Presumably this results from a great number of subsequent gene losses that have erased this signal. Likewise, the phylogenetic timing of the duplication events is also inconclusive, because duplications are common on every branch (see Figure 4). Although there is a somewhat greater number assigned to the base of vertebrates, there is no reliable way to evaluate the significance of this. In fact, even if this larger number could be found to be statistically significant, it may simply indicate that this was a period with an accelerated duplication of individual genes or multigene segments or a reduction in the rate of gene loss, rather than indicating WGD.

Conclusive evidence for 2R is seen only when data from gene families, phylogenetic trees, and genomic map position are all taken together, as has been advocated by others [21,32,43]. When examining the genomic map position of only those genes in the human genome that trace their ancestry back to a duplication event at the base of vertebrates, a clear pattern of tetra-paralogons emerges, indicating that 2R occurred at the base of vertebrates. This signal remains most clearly in 25% of the human genome that forms the largest category in the analysis shown in Figures 5 and 6, but we also find that 72% of all human genes are included in the total extent of all of the paralogons that overlap with these regions, providing the least constrained estimate of the portion of the human genome still retaining structure from the 2R. This is the outside estimate, because some portion could have as well been the result of segmental duplications of regions earlier established by WGD. This is in contrast to the pattern seen for the many other gene duplications, which generated paralogs that are predominantly arranged in tandem.

This is particularly compelling considering that this signal has survived more than 450 MY of genome rearrangements and the loss of many genes. We can imagine the effect that duplications, translocations, inversions, and deletions (and combinations thereof) would have had on this analysis: (1) Duplications would cause an increase beyond the 4-fold category; (2) translocations would decrease the 4-fold category if they are pervasive enough to clear large regions of paralogs; (3) inversions can either cause a decrease in the number of chromosomes hit by moving paralogous genes beyond the detection of the sliding window analysis or cause an increase by spreading some paralogous genes across the boundaries into adjacent segments; both of which can be exacerbated by gene translocations that blur the edges of the corresponding regions; and (4) deletions would generally increase the 3-fold chromosome category at the expense of the 4-fold category, and a deletion that occurred between the two WGDs would increase the 2-fold chromosome category. Additionally, in some cases, a few individual gene deletions or translocations may have eliminated the links between pairs of duplicated genes. Through these, and combinations of these events, the original 4-fold co-linearity established by 2R (or something less than the perfect 4-fold pattern, if these duplications were long separated) has been eroded.

These tetra-paralogons are spread across nearly all human chromosomes (Table 2). Notably, chromosome Y does not have any tetra-paralogons, perhaps due to its relatively recent origin and small number of genes, or perhaps this indicates a more rapid rate of gene movement. Chromosome 21 also has no tetra-paralogons, and Chromosome 18 has only one that is small. These chromosomes, and other regions without tetra-paralogons, could be of recent origin or they could have undergone multiple rearrangement events that would have destroyed the signal.

Although our study does not specifically address the effect that 2R has had on vertebrate evolution, we note two interesting observations. First, the vast majority of duplicated genes were subsequently deleted, indicating that relatively few genes may have been responsible for the increased complexity seen in vertebrates. Second, it is possible that many genes were loosed from constraint after the genome duplications and experienced an accelerated rate of sequence change before returning to single copy, and it is possible that this has played some role in the evolution of vertebrate complexity [44].

The mechanism of these genome duplication events, whether two separate rounds of either auto- or allo-tetraploidy or a single octoploidy, remains uncertain. We speculate that the most likely scenario is two rounds of closely spaced auto-tetraploidization events, based on the following observations. For most sets of tetra-paralogs, some pairs within the set extend over a longer region than others, indicating two distinct duplication events. If, alternatively, there had been a single octoploidy, then we would have to hypothesize multiple occasions in which two of the four descendant genomic segments lost the same sets of genes independently, which seems unlikely. The phylogenetic trees for the gene families are not consistently nested, as would be expected in the case of allo-tetraploidy or two widely spaced auto-tetraploidy events. Finally, tree topologies of genes within paralogy blocks are not always congruent, indicating that the process of gene loss and rediploidization spanned the duplication events [17].

It remains unclear to what extent such large-scale genomic events have driven macroevolutionary change versus the regular accumulation of small mutations, as is the central tenet of the classical model of evolution. We imagine that rapid and extensive evolutionary change could possibly be an emergent property of having all genes duplicated at the same time, allowing this expanded gene repertoire to evolve together, and so reach a greater level of interaction and complexity than could evolve from cumulative single gene duplications. WGDs have occurred in many lineages, including frogs [45,46], fish [41,42,47], yeast [27–30], *Arabidopsis* [27–30], and corn and several other crop species [48], all of which are being studied by modern genomics techniques. We view the broad and pervasive distribution of these tetra-paralogons in the human genome, despite the remarkably small number of genes remaining in duplicate, as robust evidence that 2R occurred at the base of Vertebrata, and anticipate that future studies will soon illuminate the roles this has played in the evolutionary success of the vertebrate lineage.

## Materials and Methods

### Obtaining chordate sequences

Sequences and gene annotations of the tunicate *Ciona intestinalis* and the pufferfish *Takifugu rubripes* were obtained from the Department of Energy Joint Genome Institute Web site at http://www.jgi.doe.gov. Sequences for *Homo sapiens* (version 19.34b.2) and *Mus musculus* (version 19.32.2) were obtained from the Ensembl project website at http://www.ensembl.org. For genes with multiple transcripts, only the longest protein sequence was taken, resulting in 15,852 *Ciona,* 37,241 fugu, 22,444 mouse, and 22,980 human genes. Table 1 shows an overview of the methods along with the numbers of genes and clusters included after each step.

### Clustering

The objective of the gene clustering is to reconstruct groups of genes such that each includes all (and only) the descendents of a single gene in the ancestral chordate. The underlying assumption is that all of the vertebrate genes in such a cluster will have a higher degree of similarity to each other than they will to their ortholog in *Ciona,* because they have arisen after the *Ciona*–vertebrate divergence by either gene duplication or lineage splitting. We conceptually translated the protein sequences for all genes, then for each *Ciona* protein sequence, the best match to any vertebrate protein was found using BLASTP [49]. Likewise, for each vertebrate protein, the best *Ciona* match was found. This list of best *Ciona–*vertebrate hits was then ordered by raw score. A graph was constructed such that each protein sequence appears at a node and the raw BLASTP scores between each pair form the weight of each edge. These sequences were then grouped by using the pairs of best hits as seeds for a single linkage clustering of the graph with the minimum edge score of the seed. This recruits to each cluster any sequence with greater similarity to the individual seed sequences than they have to each other, ensuring that genes with similarity due to a duplication before the split of *Ciona* and Vertebrata are properly apportioned into separate clusters. Any cluster that attempts to use a protein that has already been assigned is eliminated to reduce ambiguity and any cluster with greater than 100 members in a single species is eliminated. Figure 2 illustrates this clustering process.

### Phylogenetic analysis

A multiple sequence alignment for each cluster was created using ClustalW 1.81 [50]. This alignment was then trimmed by eliminating all positions with gap characters. If the remaining length of the multiple sequence alignment was less than 100 amino acids, the entire cluster was eliminated. Phylogenetic trees were constructed by using the quartet puzzling maximum likelihood method as implemented in TREE-PUZZLE 5.1 [51] using the JTT model of amino acid substitution and a gamma distribution of rates over eight rate categories with 10,000 puzzling steps used to assess reliability. Any tree whose nodes were not strictly bifurcating was eliminated. Even with strict requirements for membership in the clusters, for reliable sequence alignment, and for confidence of evolutionary analysis, we generated 6,641 gene family clusters that include 39,136 (39.7%) of 98,517 total chordate genes (see Table 1), of which 3,096 had duplicated vertebrate genes and 1,621 produced trees that are strictly bifurcating (i.e., having no polytomies).

### Identification of node types

Each node of each tree was classified in comparison to the known evolutionary relationships of the animals. For example, if the gene cluster tree contains exactly four members, and one from each animal, then the parsimonious inference is that no gene duplication occurred. In the case of a similar cluster, but where one member is missing, this is a gene loss in a single group. Gene cluster trees can show duplications specific to individual lineages by having two genes clustered together for the same animal. A gene duplication that occurred before the split of fish from tetrapods is seen as a duplicated tree of the animal relationships after their splitting from *Ciona* and, similarly, a gene duplication that occurred in tetrapods but before the split of the mouse and human lineages is seen as a duplication of the mouse–human group. Combinations of these, such as a duplication for tetrapods followed by a loss in one of the tetrapod lineages, are also seen and scored (see Figure 3).

The sorting of orthologous and paralogous relationships for each gene cluster provides an effective tool for improving the inferences of gene function by allowing annotations from well studied genomes to be transferred to the orthologous genes of other species. Inferring function from orthology is expected to be more accurate than using sequence similarity alone, since the latter tends to incorrectly associate slowly evolving paralogs. We provide a web based resource for this sorting at http://phigs.org/.

### Detecting concordantly duplicated genomic regions

An overview of the genomic distribution and patterns of paralogous gene arrangement was created by plotting the chromosomal location for all genes having a duplication before the fish–tetrapod divergence for each chromosome. We performed a sliding window analysis, looking upstream and downstream of each gene in turn, for 50 genes on each side, and asking whether paralogs generated prior to the fish–tetrapod split occur within 100 genes of each other at another genomic location. This is the most conservative approach for detecting this signal. The results are shown in Figures 5 and S2.

### Detecting paralogons and tetra-paralogons

A paralogon was defined as two chromosomal locations in the same genome that have the same set of gene pairs, allowing for a maximum of 100 unduplicated genes between. This significantly expands the set of regions that can be detected by the sliding window analysis. These were detected for the entire human genome considering separately only those paralogs generated by duplications prior to the split between fish and tetrapods versus those arising from duplications after. Each paralogon was tested for its membership in additional paralogous-region pairs. When a segment pairs with three different paralogons, we considered all six possible pairings of the four regions. If, and only if, all six possible combinations are confirmed as paralogons, the group was defined as a tetra-paralogon (see Figure 1). A minimum reconstruction of the signal of 2R remaining in the human genome is found in the extent of complete 4-fold overlap and the maximum by extending these regions to include the complete extent of all contributing paralogons. Genome and chromosome coverage were calculated by summing the number of genes that are encompassed by this more expansive reconstruction and dividing by the total number of genes.

### Inclusion of *Drosophila* genes and phylogenetic analysis

For each cluster that contained only a single gene from each of the four chordate species, the highest scoring BLASTP match to the *Drosophila melanogaster* gene set [15] was added. We then performed a multiple sequence alignment for each of these 766 sets of genes, followed by phylogenetic analysis of this concatenated data set as above. The resulting tree was rooted at the midpoint with branch lengths proportional to the amount of amino acid substitution as estimated by TREE-PUZZLE 5.1 [51].

## Supporting Information

Figure S1 Histogram of Gene Cluster Membership The numbers of genes per cluster are shown for each of the three vertebrates individually as well as for all three grouped together. There is no peak at four for any species, or at 12 as the total for all (or 16 for all, considering that there may have been a further genome duplication for fish), indicating that gene losses have been common and have eradicated this signal of genome duplications.
(30 KB JPG).

Figure S2 Plot of the Genomic Positions of Paralogous Pairs of Human Genes that Arose from Duplications Pre-dating the Fish–Tetrapod Split On the *x-*axis is each chromosome arranged from p to q telomeres. On the *y-*axis is each of the 22 human autosomes plus the X and Y chromosomes. For each query gene on the *x-*axis, a “hit” is scored if the subject chromosome contains a paralog generated by a gene duplication prior to the fish–tetrapod split. The lower portion of each panel plots the *n-*fold redundancy along the query chromosome as defined by pairs of paralogs detected in a sliding window analysis. See the Material and Methods section for details, but briefly, for every human query gene, a window was considered of 50 genes to the left and 50 genes to the right, with a “hit” obtained for the subject chromosome if it includes the early-duplicated paralogs of genes on each side of the query. The expected value of four for the 2R hypothesis is highlighted in a darker shade of blue.
(5.2 MB DOC).

Figure S3 Illustration of Whole Genome 4-Fold Paralogy The lines connect paralogous genes in the human genome that originated in duplications that occurred after the tunicate–vertebrate split but before the fish–tetrapod split. Numerals around the outside of the figure refer to human chromosome numbers.
(103 KB JPG).

Table S1 Paralogons in the Human Genome Paralogons in the human genome are defined as having two or more pairs of paralogous genes separated by no more than 100 intervening genes (see Materials and Methods). A and B in the header refer to the first and second chromosome in considered pairs. The columns labeled “Start” and “End” define the extent of each paralogon by numbered genes. The number of paralogous gene pairs defining the paralogon and the total number of genes encompassed by the region are indicated.
(344 KB DOC).

## Abbreviations

2R: two rounds of whole genome duplication
MY: million years
WGD: whole genome duplication
