# Supplementary material for: Two Rounds of Whole Genome Duplication in the Ancestral Vertebrate
Source: PLoS Biol. 2005 Sep 6;3(10):e314. doi: 10.1371/journal.pbio.0030314 (PMC1197285; doi:10.1371/journal.pbio.0030314)
Supplement: Supplementary file 2 [file pbio.0030314.st001.doc]

**Table S1**

| A | Start | End | Number  of Paralogs | Total number  of genes | B | Start | End | Number  of Paralogs | Total number  of genes |
| --- | --- | --- | --- | --- | --- | --- | --- | --- | --- |
| 1 | 1866 | 1875 | 2 | 10 | 10 | 42 | 68 | 2 | 27 |
| 1 | 2036 | 2108 | 4 | 73 | 11 | 691 | 794 | 4 | 104 |
| 1 | 1769 | 1825 | 8 | 57 | 11 | 223 | 383 | 9 | 161 |
| 1 | 1900 | 1928 | 4 | 29 | 11 | 737 | 760 | 4 | 24 |
| 1 | 1766 | 1825 | 4 | 60 | 12 | 228 | 306 | 3 | 79 |
| 1 | 1018 | 1085 | 4 | 68 | 12 | 666 | 808 | 4 | 143 |
| 1 | 1786 | 1851 | 9 | 66 | 12 | 645 | 841 | 10 | 197 |
| 1 | 47 | 114 | 4 | 68 | 12 | 70 | 90 | 4 | 21 |
| 1 | 1768 | 1799 | 3 | 32 | 12 | 5 | 20 | 3 | 16 |
| 1 | 589 | 595 | 2 | 7 | 12 | 82 | 111 | 3 | 30 |
| 1 | 397 | 497 | 2 | 101 | 13 | 13 | 56 | 2 | 44 |
| 1 | 2100 | 2113 | 5 | 14 | 14 | 660 | 670 | 4 | 11 |
| 1 | 1884 | 2091 | 27 | 208 | 14 | 251 | 656 | 27 | 406 |
| 1 | 1203 | 1429 | 24 | 227 | 15 | 236 | 656 | 25 | 421 |
| 1 | 1952 | 2084 | 5 | 133 | 15 | 86 | 184 | 4 | 99 |
| 1 | 1161 | 1223 | 2 | 63 | 15 | 528 | 530 | 2 | 3 |
| 1 | 195 | 385 | 5 | 191 | 16 | 688 | 808 | 4 | 121 |
| 1 | 27 | 114 | 5 | 88 | 17 | 106 | 194 | 5 | 89 |
| 1 | 327 | 433 | 4 | 107 | 18 | 10 | 35 | 4 | 26 |
| 1 | 1151 | 1429 | 12 | 279 | 19 | 9 | 229 | 12 | 221 |
| 1 | 610 | 977 | 40 | 368 | 19 | 5 | 529 | 43 | 525 |
| 1 | 1623 | 1686 | 3 | 64 | 19 | 326 | 406 | 3 | 81 |
| 1 | 1435 | 1532 | 4 | 98 | 19 | 856 | 933 | 3 | 78 |
| 1 | 1325 | 1369 | 3 | 45 | 19 | 522 | 567 | 3 | 46 |
| 1 | 1922 | 2108 | 9 | 187 | 19 | 755 | 831 | 9 | 77 |
| 1 | 360 | 552 | 5 | 193 | 2 | 44 | 146 | 5 | 103 |
| 1 | 316 | 501 | 11 | 186 | 20 | 236 | 609 | 11 | 374 |
| 1 | 333 | 345 | 4 | 13 | 21 | 111 | 126 | 3 | 16 |
| 1 | 994 | 1020 | 4 | 27 | 3 | 302 | 461 | 4 | 160 |
| 1 | 27 | 169 | 16 | 143 | 3 | 816 | 1101 | 19 | 286 |
| 1 | 1768 | 1866 | 5 | 99 | 3 | 296 | 416 | 5 | 121 |
| 1 | 1785 | 1875 | 4 | 91 | 3 | 1 | 32 | 4 | 32 |
| 1 | 286 | 287 | 2 | 2 | 3 | 1033 | 1036 | 2 | 4 |
| 1 | 1348 | 1413 | 7 | 66 | 5 | 361 | 536 | 7 | 176 |
| 1 | 605 | 810 | 15 | 206 | 5 | 166 | 389 | 15 | 224 |
| 1 | 1119 | 1218 | 3 | 100 | 5 | 110 | 192 | 3 | 83 |
| 1 | 1376 | 1395 | 2 | 20 | 5 | 120 | 201 | 2 | 82 |
| 1 | 311 | 563 | 31 | 253 | 6 | 51 | 1037 | 34 | 987 |
| 1 | 230 | 233 | 2 | 4 | 6 | 1065 | 1068 | 2 | 4 |
| 1 | 1544 | 1713 | 8 | 170 | 6 | 288 | 405 | 7 | 118 |
| 1 | 828 | 919 | 2 | 92 | 6 | 338 | 403 | 2 | 66 |
| 1 | 1189 | 1195 | 4 | 7 | 6 | 133 | 133 | 1 | 1 |
| 1 | 1827 | 1828 | 2 | 2 | 7 | 732 | 738 | 2 | 7 |
| 1 | 230 | 233 | 2 | 4 | 7 | 501 | 501 | 1 | 1 |
| 1 | 998 | 1050 | 4 | 53 | 7 | 715 | 822 | 4 | 108 |
| 1 | 992 | 1068 | 4 | 77 | 7 | 491 | 512 | 4 | 22 |
| 1 | 359 | 546 | 7 | 188 | 8 | 603 | 743 | 5 | 141 |
| 1 | 323 | 463 | 7 | 141 | 8 | 17 | 417 | 8 | 401 |
| 1 | 1463 | 1756 | 24 | 294 | 9 | 546 | 841 | 25 | 296 |
| 1 | 1203 | 1239 | 2 | 37 | 9 | 429 | 435 | 2 | 7 |
| 1 | 838 | 981 | 17 | 144 | 9 | 477 | 767 | 17 | 291 |
| 1 | 602 | 788 | 11 | 187 | 9 | 19 | 129 | 11 | 111 |
| 1 | 1352 | 1398 | 3 | 47 | 9 | 329 | 363 | 3 | 35 |
| 1 | 1161 | 1343 | 9 | 183 | 9 | 146 | 312 | 9 | 167 |
| 1 | 631 | 858 | 5 | 228 | 9 | 280 | 407 | 5 | 128 |
| 1 | 497 | 522 | 2 | 26 | X | 330 | 370 | 2 | 41 |
| 1 | 1587 | 1597 | 2 | 11 | X | 612 | 711 | 2 | 100 |
| 1 | 1768 | 1866 | 4 | 99 | X | 208 | 307 | 4 | 100 |
| 1 | 998 | 1050 | 2 | 53 | X | 238 | 248 | 2 | 11 |
| 10 | 166 | 466 | 10 | 301 | 12 | 604 | 617 | 3 | 14 |
| 10 | 3 | 17 | 3 | 15 | 12 | 351 | 413 | 3 | 63 |
| 10 | 174 | 182 | 2 | 9 | 12 | 414 | 476 | 2 | 63 |
| 10 | 89 | 174 | 4 | 86 | 17 | 734 | 770 | 4 | 37 |
| 10 | 107 | 132 | 5 | 26 | 17 | 525 | 539 | 5 | 15 |
| 10 | 444 | 758 | 17 | 315 | 2 | 377 | 596 | 16 | 220 |
| 10 | 101 | 174 | 8 | 74 | 2 | 866 | 976 | 8 | 111 |
| 10 | 254 | 341 | 7 | 88 | 2 | 360 | 495 | 6 | 136 |
| 10 | 139 | 186 | 6 | 48 | 2 | 1168 | 1194 | 6 | 27 |
| 10 | 492 | 771 | 6 | 280 | 20 | 49 | 77 | 5 | 29 |
| 10 | 3 | 17 | 3 | 15 | 21 | 215 | 259 | 3 | 45 |
| 10 | 29 | 80 | 5 | 52 | 3 | 296 | 480 | 5 | 185 |
| 10 | 65 | 174 | 3 | 110 | 3 | 687 | 730 | 3 | 44 |
| 10 | 214 | 696 | 28 | 483 | 4 | 341 | 574 | 29 | 234 |
| 10 | 509 | 804 | 21 | 296 | 4 | 25 | 828 | 21 | 804 |
| 10 | 401 | 809 | 17 | 409 | 5 | 762 | 952 | 17 | 191 |
| 10 | 296 | 380 | 6 | 85 | 5 | 562 | 649 | 6 | 88 |
| 10 | 206 | 360 | 7 | 155 | 5 | 916 | 987 | 5 | 72 |
| 10 | 634 | 807 | 5 | 174 | 5 | 587 | 728 | 5 | 142 |
| 10 | 549 | 771 | 5 | 223 | 7 | 281 | 302 | 4 | 22 |
| 10 | 225 | 466 | 8 | 242 | 7 | 1015 | 1015 | 1 | 1 |
| 10 | 265 | 426 | 5 | 162 | 7 | 286 | 295 | 4 | 10 |
| 10 | 504 | 814 | 20 | 311 | 8 | 74 | 298 | 20 | 225 |
| 10 | 408 | 416 | 4 | 9 | 8 | 257 | 301 | 4 | 45 |
| 10 | 203 | 315 | 3 | 113 | 8 | 140 | 174 | 3 | 35 |
| 10 | 285 | 300 | 2 | 16 | 8 | 294 | 300 | 2 | 7 |
| 10 | 528 | 613 | 2 | 86 | 9 | 222 | 282 | 2 | 61 |
| 10 | 42 | 82 | 2 | 41 | X | 228 | 307 | 2 | 80 |
| 11 | 294 | 345 | 7 | 52 | 12 | 688 | 803 | 7 | 116 |
| 11 | 222 | 310 | 10 | 89 | 12 | 228 | 291 | 8 | 64 |
| 11 | 651 | 665 | 3 | 15 | 12 | 674 | 681 | 3 | 8 |
| 11 | 79 | 91 | 3 | 13 | 12 | 689 | 709 | 3 | 21 |
| 11 | 5 | 76 | 4 | 72 | 12 | 816 | 846 | 3 | 31 |
| 11 | 234 | 270 | 2 | 37 | 12 | 27 | 28 | 2 | 2 |
| 11 | 24 | 41 | 3 | 18 | 12 | 222 | 285 | 3 | 64 |
| 11 | 861 | 862 | 2 | 2 | 12 | 888 | 892 | 2 | 5 |
| 11 | 623 | 845 | 20 | 223 | 14 | 177 | 684 | 20 | 508 |
| 11 | 821 | 822 | 2 | 2 | 14 | 653 | 681 | 2 | 29 |
| 11 | 734 | 775 | 4 | 42 | 15 | 117 | 184 | 4 | 68 |
| 11 | 871 | 1030 | 5 | 160 | 17 | 62 | 148 | 5 | 87 |
| 11 | 882 | 885 | 2 | 4 | 17 | 342 | 344 | 2 | 3 |
| 11 | 691 | 825 | 5 | 135 | 19 | 742 | 831 | 5 | 90 |
| 11 | 680 | 798 | 3 | 119 | 19 | 939 | 1011 | 3 | 73 |
| 11 | 278 | 448 | 5 | 171 | 19 | 1057 | 1123 | 5 | 67 |
| 11 | 1248 | 1380 | 3 | 133 | 19 | 661 | 698 | 3 | 38 |
| 11 | 910 | 912 | 2 | 3 | 19 | 1073 | 1130 | 2 | 58 |
| 11 | 1400 | 1401 | 2 | 2 | 19 | 1157 | 1157 | 1 | 1 |
| 11 | 679 | 845 | 9 | 167 | 2 | 186 | 316 | 9 | 131 |
| 11 | 936 | 986 | 3 | 51 | 2 | 1303 | 1377 | 3 | 75 |
| 11 | 952 | 953 | 2 | 2 | 2 | 796 | 824 | 3 | 29 |
| 11 | 725 | 802 | 4 | 78 | 20 | 110 | 210 | 5 | 101 |
| 11 | 1174 | 1377 | 8 | 204 | 21 | 144 | 198 | 8 | 55 |
| 11 | 424 | 443 | 2 | 20 | 22 | 449 | 522 | 2 | 74 |
| 11 | 606 | 613 | 3 | 8 | 22 | 251 | 254 | 2 | 4 |
| 11 | 892 | 912 | 2 | 21 | 22 | 512 | 524 | 2 | 13 |
| 11 | 223 | 253 | 2 | 31 | 22 | 446 | 513 | 2 | 68 |
| 11 | 819 | 863 | 2 | 45 | 22 | 239 | 249 | 2 | 11 |
| 11 | 1254 | 1401 | 6 | 148 | 3 | 553 | 634 | 5 | 82 |
| 11 | 953 | 977 | 2 | 25 | 3 | 787 | 789 | 2 | 3 |
| 11 | 999 | 1030 | 2 | 32 | 3 | 1124 | 1130 | 2 | 7 |
| 11 | 420 | 479 | 2 | 60 | 3 | 828 | 845 | 2 | 18 |
| 11 | 926 | 1017 | 5 | 92 | 4 | 649 | 799 | 4 | 151 |
| 11 | 937 | 965 | 3 | 29 | 4 | 173 | 210 | 3 | 38 |
| 11 | 1139 | 1148 | 3 | 10 | 4 | 648 | 727 | 3 | 80 |
| 11 | 105 | 193 | 2 | 89 | 4 | 171 | 207 | 2 | 37 |
| 11 | 938 | 1017 | 2 | 80 | 5 | 848 | 939 | 3 | 92 |
| 11 | 443 | 458 | 2 | 16 | 6 | 417 | 457 | 2 | 41 |
| 11 | 1162 | 1163 | 2 | 2 | 6 | 994 | 1048 | 2 | 55 |
| 11 | 1037 | 1042 | 2 | 6 | 6 | 708 | 709 | 2 | 2 |
| 11 | 250 | 270 | 2 | 21 | 6 | 427 | 432 | 2 | 6 |
| 11 | 737 | 902 | 3 | 166 | 6 | 475 | 554 | 5 | 80 |
| 11 | 361 | 430 | 3 | 70 | 7 | 808 | 872 | 4 | 65 |
| 11 | 1162 | 1163 | 2 | 2 | X | 330 | 333 | 2 | 4 |
| 11 | 1068 | 1098 | 2 | 31 | X | 761 | 764 | 2 | 4 |
| 11 | 978 | 1030 | 2 | 53 | X | 355 | 360 | 2 | 6 |
| 11 | 999 | 1017 | 2 | 19 | X | 559 | 637 | 2 | 79 |
| 12 | 483 | 494 | 2 | 12 | 13 | 281 | 336 | 2 | 56 |
| 12 | 411 | 507 | 2 | 97 | 15 | 108 | 111 | 2 | 4 |
| 12 | 236 | 239 | 4 | 4 | 15 | 632 | 632 | 1 | 1 |
| 12 | 870 | 1063 | 6 | 194 | 16 | 409 | 486 | 6 | 78 |
| 12 | 198 | 199 | 2 | 2 | 16 | 304 | 304 | 1 | 1 |
| 12 | 347 | 604 | 8 | 258 | 17 | 687 | 827 | 8 | 141 |
| 12 | 477 | 598 | 4 | 122 | 17 | 541 | 641 | 4 | 101 |
| 12 | 867 | 1069 | 8 | 203 | 17 | 271 | 510 | 8 | 240 |
| 12 | 70 | 111 | 4 | 42 | 17 | 106 | 194 | 3 | 89 |
| 12 | 935 | 936 | 2 | 2 | 17 | 922 | 928 | 2 | 7 |
| 12 | 988 | 1023 | 2 | 36 | 17 | 76 | 131 | 2 | 56 |
| 12 | 198 | 199 | 2 | 2 | 17 | 1028 | 1028 | 1 | 1 |
| 12 | 245 | 255 | 2 | 11 | 19 | 1052 | 1061 | 2 | 10 |
| 12 | 343 | 627 | 24 | 285 | 2 | 864 | 1250 | 26 | 387 |
| 12 | 377 | 418 | 5 | 42 | 2 | 1125 | 1289 | 5 | 165 |
| 12 | 73 | 77 | 2 | 5 | 2 | 1353 | 1450 | 2 | 98 |
| 12 | 351 | 413 | 2 | 63 | 21 | 215 | 259 | 2 | 45 |
| 12 | 314 | 323 | 4 | 10 | 3 | 388 | 489 | 4 | 102 |
| 12 | 78 | 111 | 6 | 34 | 3 | 920 | 1078 | 5 | 159 |
| 12 | 13 | 20 | 4 | 8 | 3 | 415 | 424 | 4 | 10 |
| 12 | 420 | 838 | 20 | 419 | 3 | 70 | 432 | 20 | 363 |
| 12 | 766 | 786 | 2 | 21 | 3 | 757 | 846 | 2 | 90 |
| 12 | 2 | 4 | 3 | 3 | 3 | 72 | 84 | 2 | 13 |
| 12 | 264 | 305 | 2 | 42 | 3 | 11 | 29 | 2 | 19 |
| 12 | 236 | 239 | 4 | 4 | 3 | 789 | 789 | 1 | 1 |
| 12 | 264 | 305 | 4 | 42 | 6 | 408 | 458 | 4 | 51 |
| 12 | 677 | 794 | 3 | 118 | 6 | 416 | 499 | 3 | 84 |
| 12 | 22 | 38 | 5 | 17 | 6 | 432 | 505 | 5 | 74 |
| 12 | 894 | 896 | 2 | 3 | 7 | 675 | 676 | 2 | 2 |
| 12 | 1003 | 1063 | 5 | 61 | 7 | 417 | 462 | 5 | 46 |
| 12 | 762 | 827 | 2 | 66 | 7 | 281 | 281 | 1 | 1 |
| 12 | 483 | 582 | 5 | 100 | 7 | 127 | 334 | 5 | 208 |
| 12 | 377 | 403 | 4 | 27 | 7 | 1010 | 1049 | 4 | 40 |
| 12 | 343 | 347 | 2 | 5 | 7 | 99 | 133 | 2 | 35 |
| 12 | 297 | 298 | 2 | 2 | 9 | 383 | 388 | 2 | 6 |
| 12 | 434 | 436 | 2 | 3 | 9 | 445 | 449 | 2 | 5 |
| 12 | 298 | 337 | 5 | 40 | X | 220 | 301 | 3 | 82 |
| 12 | 754 | 786 | 2 | 33 | X | 699 | 798 | 2 | 100 |
| 12 | 2 | 20 | 3 | 19 | X | 249 | 290 | 3 | 42 |
| 13 | 175 | 343 | 9 | 169 | 2 | 1308 | 1445 | 9 | 138 |
| 13 | 186 | 374 | 8 | 189 | 3 | 805 | 1015 | 8 | 211 |
| 13 | 52 | 73 | 3 | 22 | 4 | 215 | 265 | 3 | 51 |
| 13 | 90 | 139 | 7 | 50 | 4 | 595 | 731 | 7 | 137 |
| 13 | 244 | 257 | 3 | 14 | 4 | 181 | 209 | 3 | 29 |
| 13 | 1 | 121 | 4 | 121 | 5 | 500 | 616 | 4 | 117 |
| 13 | 236 | 271 | 4 | 36 | 5 | 697 | 723 | 4 | 27 |
| 13 | 27 | 138 | 3 | 112 | 6 | 997 | 1022 | 3 | 26 |
| 13 | 33 | 78 | 5 | 46 | 8 | 122 | 128 | 5 | 7 |
| 13 | 215 | 245 | 7 | 31 | X | 409 | 449 | 6 | 41 |
| 13 | 284 | 363 | 12 | 80 | X | 543 | 723 | 12 | 181 |
| 13 | 13 | 78 | 6 | 66 | X | 328 | 398 | 6 | 71 |
| 13 | 239 | 242 | 2 | 4 | Y | 5 | 5 | 1 | 1 |
| 14 | 49 | 123 | 6 | 75 | 16 | 642 | 873 | 6 | 232 |
| 14 | 105 | 106 | 2 | 2 | 17 | 238 | 243 | 6 | 6 |
| 14 | 122 | 124 | 2 | 3 | 17 | 673 | 673 | 1 | 1 |
| 14 | 156 | 397 | 10 | 242 | 19 | 970 | 1015 | 9 | 46 |
| 14 | 382 | 419 | 5 | 38 | 19 | 742 | 820 | 5 | 79 |
| 14 | 243 | 275 | 2 | 33 | 19 | 782 | 838 | 3 | 57 |
| 14 | 614 | 649 | 3 | 36 | 19 | 948 | 967 | 3 | 20 |
| 14 | 159 | 684 | 24 | 526 | 2 | 208 | 356 | 25 | 149 |
| 14 | 81 | 287 | 20 | 207 | 20 | 105 | 532 | 19 | 428 |
| 14 | 677 | 680 | 2 | 4 | 20 | 120 | 124 | 2 | 5 |
| 14 | 433 | 509 | 3 | 77 | 20 | 135 | 209 | 3 | 75 |
| 14 | 259 | 260 | 2 | 2 | 5 | 167 | 167 | 1 | 1 |
| 14 | 253 | 285 | 2 | 33 | 6 | 1099 | 1112 | 2 | 14 |
| 14 | 225 | 307 | 2 | 83 | 6 | 466 | 480 | 2 | 15 |
| 14 | 632 | 634 | 2 | 3 | 6 | 521 | 555 | 2 | 35 |
| 14 | 27 | 131 | 6 | 105 | 8 | 317 | 499 | 6 | 183 |
| 14 | 422 | 424 | 3 | 3 | 9 | 458 | 458 | 1 | 1 |
| 15 | 281 | 570 | 12 | 290 | 18 | 159 | 227 | 11 | 69 |
| 15 | 236 | 622 | 23 | 387 | 19 | 9 | 269 | 24 | 261 |
| 15 | 86 | 173 | 5 | 88 | 19 | 711 | 848 | 5 | 138 |
| 15 | 472 | 551 | 2 | 80 | 19 | 489 | 555 | 2 | 67 |
| 15 | 117 | 178 | 3 | 62 | 2 | 186 | 218 | 3 | 33 |
| 15 | 454 | 455 | 2 | 2 | 2 | 219 | 219 | 1 | 1 |
| 15 | 129 | 131 | 2 | 3 | 20 | 110 | 113 | 3 | 4 |
| 15 | 200 | 201 | 2 | 2 | 20 | 349 | 349 | 1 | 1 |
| 15 | 24 | 43 | 3 | 20 | 4 | 228 | 237 | 3 | 10 |
| 15 | 434 | 515 | 4 | 82 | 5 | 119 | 215 | 4 | 97 |
| 15 | 459 | 656 | 12 | 198 | 5 | 356 | 520 | 12 | 165 |
| 15 | 23 | 43 | 3 | 21 | 5 | 813 | 838 | 3 | 26 |
| 15 | 108 | 250 | 6 | 143 | 5 | 381 | 547 | 5 | 167 |
| 15 | 70 | 93 | 2 | 24 | 5 | 20 | 112 | 2 | 93 |
| 15 | 200 | 201 | 2 | 2 | 6 | 42 | 42 | 1 | 1 |
| 15 | 184 | 196 | 2 | 13 | 6 | 512 | 553 | 2 | 42 |
| 15 | 257 | 357 | 3 | 101 | 8 | 122 | 236 | 3 | 115 |
| 15 | 53 | 670 | 22 | 618 | 9 | 78 | 429 | 25 | 352 |
| 16 | 6 | 450 | 19 | 445 | 17 | 904 | 1177 | 20 | 274 |
| 16 | 65 | 88 | 3 | 24 | 17 | 836 | 853 | 3 | 18 |
| 16 | 26 | 37 | 3 | 12 | 17 | 443 | 449 | 3 | 7 |
| 16 | 234 | 278 | 3 | 45 | 17 | 221 | 329 | 4 | 109 |
| 16 | 439 | 497 | 4 | 59 | 17 | 379 | 487 | 4 | 109 |
| 16 | 409 | 413 | 2 | 5 | 17 | 78 | 85 | 3 | 8 |
| 16 | 919 | 933 | 3 | 15 | 18 | 41 | 64 | 2 | 24 |
| 16 | 687 | 737 | 2 | 51 | 18 | 156 | 251 | 2 | 96 |
| 16 | 292 | 363 | 3 | 72 | 19 | 1213 | 1223 | 4 | 11 |
| 16 | 516 | 524 | 2 | 9 | 2 | 745 | 745 | 1 | 1 |
| 16 | 747 | 889 | 5 | 143 | 20 | 228 | 379 | 5 | 152 |
| 16 | 581 | 715 | 4 | 135 | 20 | 444 | 495 | 4 | 52 |
| 16 | 551 | 642 | 3 | 92 | 20 | 231 | 336 | 3 | 106 |
| 16 | 63 | 248 | 10 | 186 | 22 | 294 | 432 | 10 | 139 |
| 16 | 353 | 508 | 5 | 156 | 22 | 217 | 335 | 6 | 119 |
| 16 | 450 | 452 | 2 | 3 | 22 | 110 | 111 | 2 | 2 |
| 16 | 776 | 860 | 4 | 85 | 3 | 768 | 845 | 2 | 78 |
| 16 | 663 | 664 | 2 | 2 | 3 | 148 | 149 | 2 | 2 |
| 16 | 562 | 563 | 2 | 2 | 3 | 1023 | 1023 | 1 | 1 |
| 16 | 574 | 715 | 5 | 142 | 5 | 9 | 43 | 5 | 35 |
| 16 | 878 | 881 | 2 | 4 | 6 | 7 | 8 | 2 | 2 |
| 16 | 615 | 684 | 3 | 70 | 7 | 232 | 241 | 3 | 10 |
| 16 | 475 | 486 | 3 | 12 | 7 | 347 | 423 | 3 | 77 |
| 16 | 733 | 759 | 2 | 27 | 8 | 619 | 680 | 2 | 62 |
| 16 | 618 | 889 | 8 | 272 | 8 | 434 | 516 | 8 | 83 |
| 16 | 531 | 554 | 2 | 24 | 8 | 723 | 776 | 2 | 54 |
| 16 | 447 | 476 | 2 | 30 | 9 | 461 | 463 | 2 | 3 |
| 16 | 776 | 813 | 3 | 38 | X | 190 | 190 | 1 | 1 |
| 16 | 516 | 524 | 2 | 9 | X | 343 | 343 | 1 | 1 |
| 17 | 976 | 978 | 3 | 3 | 19 | 1213 | 1215 | 3 | 3 |
| 17 | 453 | 551 | 3 | 99 | 2 | 1217 | 1289 | 3 | 73 |
| 17 | 458 | 938 | 7 | 481 | 2 | 888 | 978 | 6 | 91 |
| 17 | 658 | 1009 | 9 | 352 | 2 | 1147 | 1294 | 7 | 148 |
| 17 | 743 | 828 | 8 | 86 | 2 | 861 | 1033 | 8 | 173 |
| 17 | 238 | 243 | 6 | 6 | 20 | 293 | 293 | 1 | 1 |
| 17 | 15 | 42 | 4 | 28 | 22 | 86 | 261 | 4 | 176 |
| 17 | 251 | 299 | 2 | 49 | 22 | 397 | 429 | 2 | 33 |
| 17 | 861 | 1203 | 16 | 343 | 22 | 112 | 432 | 15 | 321 |
| 17 | 347 | 514 | 7 | 168 | 22 | 136 | 253 | 7 | 118 |
| 17 | 98 | 185 | 9 | 88 | 3 | 973 | 1130 | 9 | 158 |
| 17 | 541 | 735 | 10 | 195 | 3 | 112 | 204 | 10 | 93 |
| 17 | 104 | 184 | 3 | 81 | 3 | 801 | 872 | 3 | 72 |
| 17 | 687 | 822 | 6 | 136 | 7 | 94 | 206 | 6 | 113 |
| 17 | 825 | 964 | 6 | 140 | 7 | 565 | 665 | 6 | 101 |
| 17 | 544 | 630 | 6 | 87 | 7 | 222 | 334 | 6 | 113 |
| 17 | 1125 | 1213 | 4 | 89 | 7 | 42 | 72 | 4 | 31 |
| 17 | 938 | 948 | 3 | 11 | 7 | 995 | 1021 | 3 | 27 |
| 17 | 971 | 990 | 2 | 20 | 7 | 39 | 41 | 2 | 3 |
| 17 | 650 | 713 | 4 | 64 | 7 | 878 | 1014 | 4 | 137 |
| 17 | 200 | 203 | 3 | 4 | 9 | 387 | 387 | 1 | 1 |
| 17 | 90 | 166 | 3 | 77 | X | 355 | 367 | 3 | 13 |
| 18 | 159 | 204 | 4 | 46 | 19 | 62 | 141 | 4 | 80 |
| 18 | 87 | 143 | 5 | 57 | 2 | 55 | 137 | 4 | 83 |
| 18 | 10 | 35 | 4 | 26 | 20 | 321 | 370 | 4 | 50 |
| 18 | 47 | 114 | 5 | 68 | 20 | 481 | 567 | 5 | 87 |
| 18 | 143 | 152 | 2 | 10 | 20 | 253 | 348 | 2 | 96 |
| 18 | 287 | 295 | 3 | 9 | 20 | 493 | 497 | 3 | 5 |
| 18 | 10 | 35 | 5 | 26 | 6 | 823 | 904 | 5 | 82 |
| 18 | 91 | 190 | 2 | 100 | 8 | 143 | 214 | 2 | 72 |
| 19 | 939 | 1015 | 9 | 77 | 2 | 186 | 330 | 9 | 145 |
| 19 | 774 | 823 | 2 | 50 | 20 | 113 | 142 | 2 | 30 |
| 19 | 628 | 650 | 2 | 23 | 20 | 497 | 564 | 2 | 68 |
| 19 | 970 | 1008 | 2 | 39 | 20 | 182 | 188 | 2 | 7 |
| 19 | 1033 | 1110 | 2 | 78 | 22 | 328 | 387 | 2 | 60 |
| 19 | 1214 | 1215 | 2 | 2 | 22 | 312 | 312 | 1 | 1 |
| 19 | 381 | 559 | 9 | 179 | 5 | 240 | 495 | 8 | 256 |
| 19 | 56 | 256 | 6 | 201 | 5 | 486 | 547 | 6 | 62 |
| 19 | 5 | 100 | 4 | 96 | 5 | 201 | 273 | 4 | 73 |
| 19 | 426 | 452 | 2 | 27 | 6 | 307 | 380 | 2 | 74 |
| 19 | 297 | 323 | 2 | 27 | 6 | 338 | 386 | 2 | 49 |
| 19 | 620 | 622 | 2 | 3 | 8 | 519 | 523 | 2 | 5 |
| 19 | 9 | 415 | 21 | 407 | 9 | 7 | 369 | 20 | 363 |
| 19 | 406 | 512 | 5 | 107 | 9 | 669 | 841 | 5 | 173 |
| 19 | 205 | 326 | 11 | 122 | 9 | 605 | 834 | 11 | 230 |
| 19 | 397 | 488 | 2 | 92 | 9 | 483 | 487 | 2 | 5 |
| 2 | 110 | 130 | 2 | 21 | 20 | 252 | 253 | 2 | 2 |
| 2 | 900 | 920 | 2 | 21 | 21 | 144 | 228 | 2 | 85 |
| 2 | 796 | 920 | 3 | 125 | 3 | 694 | 787 | 2 | 94 |
| 2 | 871 | 1117 | 7 | 247 | 3 | 100 | 190 | 7 | 91 |
| 2 | 1146 | 1253 | 5 | 108 | 3 | 172 | 272 | 5 | 101 |
| 2 | 769 | 770 | 2 | 2 | 3 | 157 | 158 | 2 | 2 |
| 2 | 567 | 638 | 5 | 72 | 4 | 130 | 273 | 5 | 144 |
| 2 | 618 | 643 | 2 | 26 | 4 | 651 | 654 | 2 | 4 |
| 2 | 688 | 689 | 2 | 2 | 4 | 396 | 396 | 1 | 1 |
| 2 | 382 | 443 | 2 | 62 | 4 | 49 | 102 | 2 | 54 |
| 2 | 360 | 473 | 6 | 114 | 4 | 343 | 538 | 6 | 196 |
| 2 | 413 | 495 | 4 | 83 | 5 | 587 | 639 | 4 | 53 |
| 2 | 688 | 689 | 2 | 2 | 5 | 573 | 573 | 1 | 1 |
| 2 | 366 | 643 | 10 | 278 | 5 | 831 | 989 | 11 | 159 |
| 2 | 28 | 31 | 2 | 4 | 6 | 107 | 110 | 2 | 4 |
| 2 | 745 | 752 | 2 | 8 | 7 | 990 | 1041 | 3 | 52 |
| 2 | 1260 | 1389 | 5 | 130 | 7 | 1010 | 1057 | 5 | 48 |
| 2 | 992 | 1161 | 11 | 170 | 7 | 94 | 269 | 11 | 176 |
| 2 | 1321 | 1426 | 3 | 106 | 7 | 624 | 647 | 3 | 24 |
| 2 | 983 | 991 | 4 | 9 | 7 | 570 | 576 | 4 | 7 |
| 2 | 388 | 582 | 4 | 195 | 8 | 145 | 227 | 4 | 83 |
| 2 | 32 | 155 | 12 | 124 | 8 | 547 | 742 | 12 | 196 |
| 2 | 901 | 908 | 2 | 8 | 9 | 445 | 449 | 2 | 5 |
| 2 | 762 | 763 | 2 | 2 | 9 | 497 | 498 | 2 | 2 |
| 2 | 1313 | 1445 | 5 | 133 | X | 543 | 670 | 6 | 128 |
| 2 | 1377 | 1417 | 2 | 41 | X | 327 | 355 | 2 | 29 |
| 2 | 634 | 689 | 4 | 56 | X | 524 | 599 | 3 | 76 |
| 20 | 86 | 90 | 2 | 5 | 4 | 355 | 438 | 2 | 84 |
| 20 | 73 | 87 | 2 | 15 | 5 | 632 | 646 | 2 | 15 |
| 20 | 41 | 412 | 5 | 372 | 6 | 42 | 111 | 3 | 70 |
| 20 | 523 | 603 | 3 | 81 | 6 | 61 | 676 | 5 | 616 |
| 20 | 321 | 622 | 9 | 302 | 6 | 823 | 976 | 9 | 154 |
| 20 | 283 | 480 | 20 | 198 | 8 | 317 | 775 | 22 | 459 |
| 20 | 10 | 25 | 2 | 16 | 8 | 602 | 647 | 2 | 46 |
| 21 | 124 | 126 | 2 | 3 | 6 | 569 | 574 | 2 | 6 |
| 21 | 91 | 101 | 2 | 11 | 6 | 1029 | 1039 | 2 | 11 |
| 22 | 484 | 503 | 4 | 20 | 3 | 30 | 65 | 4 | 36 |
| 22 | 449 | 511 | 4 | 63 | 6 | 417 | 443 | 5 | 27 |
| 22 | 327 | 368 | 5 | 42 | 7 | 6 | 75 | 5 | 70 |
| 3 | 11 | 65 | 4 | 55 | 6 | 408 | 453 | 4 | 46 |
| 3 | 674 | 676 | 2 | 3 | 6 | 851 | 852 | 2 | 2 |
| 3 | 1 | 26 | 5 | 26 | 7 | 708 | 755 | 5 | 48 |
| 3 | 331 | 347 | 2 | 17 | 7 | 497 | 501 | 2 | 5 |
| 3 | 1054 | 1057 | 2 | 4 | 7 | 106 | 148 | 2 | 43 |
| 3 | 314 | 483 | 10 | 170 | 7 | 715 | 811 | 10 | 97 |
| 3 | 800 | 1134 | 7 | 335 | 7 | 625 | 669 | 7 | 45 |
| 3 | 859 | 860 | 2 | 2 | 8 | 403 | 403 | 1 | 1 |
| 3 | 800 | 1015 | 11 | 216 | X | 187 | 743 | 11 | 557 |
| 3 | 986 | 989 | 2 | 4 | X | 26 | 40 | 2 | 15 |
| 3 | 296 | 466 | 7 | 171 | X | 197 | 309 | 7 | 113 |
| 3 | 414 | 442 | 2 | 29 | X | 828 | 829 | 2 | 2 |
| 3 | 64 | 72 | 3 | 9 | X | 204 | 290 | 3 | 87 |
| 4 | 351 | 836 | 19 | 486 | 5 | 557 | 791 | 19 | 235 |
| 4 | 31 | 239 | 18 | 209 | 5 | 633 | 930 | 17 | 298 |
| 4 | 265 | 541 | 8 | 277 | 5 | 913 | 993 | 9 | 81 |
| 4 | 647 | 651 | 3 | 5 | 5 | 975 | 983 | 3 | 9 |
| 4 | 564 | 570 | 4 | 7 | 5 | 762 | 767 | 3 | 6 |
| 4 | 506 | 564 | 2 | 59 | 7 | 288 | 297 | 2 | 10 |
| 4 | 746 | 754 | 2 | 9 | 7 | 283 | 302 | 2 | 20 |
